# Response of a Coastal Microbial Community to Olivine Addition in the Muping Marine Ranch, Yantai

**DOI:** 10.3389/fmicb.2021.805361

**Published:** 2022-02-10

**Authors:** Hongwei Ren, Yubin Hu, Jihua Liu, Zhe Zhang, Liang Mou, Yanning Pan, Qiang Zheng, Gang Li, Nianzhi Jiao

**Affiliations:** ^1^Institute of Marine Science and Technology, Shandong University, Qingdao, China; ^2^Joint Laboratory for Ocean Research and Education at Dalhousie University, Shandong University, Qingdao, China; ^3^Joint Laboratory for Ocean Research and Education at Dalhousie University, Xiamen University, Xiamen, China; ^4^Southern Marine Science and Engineering Guangdong Laboratory, Zhuhai, China; ^5^School of Earth Science and Resources, Chang’an University, Xi’an, China; ^6^College of Geology and Environment, Xi’an University of Science and Technology, Xi’an, China; ^7^Institute of Marine Microbes and Ecospheres, Xiamen University, Xiamen, China; ^8^State Key Laboratory of Marine Environmental Science and College of Ocean and Earth Sciences, Fujian Key Laboratory of Marine Carbon Sequestration, Xiamen University, Xiamen, China; ^9^Key Laboratory of Tropical Marine Bio-Resources and Ecology, South China Sea Institute of Oceanology, Chinese Academy of Sciences, Guangzhou, China

**Keywords:** CO_2_ sequestration, silicate mineral dissolution, seawater alkalinity, enhanced weathering, bacterial community

## Abstract

Spreading olivine powder in seawater to enhance alkalinity through weathering reactions has been proposed as a potential solution to control atmospheric CO_2_ concentration. Attention has usually been paid to the chemical properties of seawater after the addition of olivine within lab and modeling studies. However, both microbial acclimation and evolution in such manipulated natural environments are often overlooked, yet they are of great importance for understanding the biological consequences of whether olivine addition is a feasible approach to mitigating climate change. In this study, an olivine addition experiment was conducted to investigate variation in bacterial diversity and community composition in the surface and bottom seawater of a representative marine ranch area in the Muping, Yantai. The results show that the composition of the particle-attached microbial community was particularly affected by the application of olivine. The relative abundance of biofilm-forming microbes in particle-attached fraction increased after the addition of olivine, while no significant variation in the free-living bacterial community was observed. Our study suggests that olivine addition would reshape the bacterial community structure, especially in particle-attached microenvironments. Therefore, the risk evaluation of alkalinity enhancement should be further studied before its large-scale application as a potential ocean geoengineering plan.

## Introduction

Massive fossil fuel combustion has contributed to a significant increase in atmospheric CO_2_ content, resulting in an increase in *p*CO_2_ from 280 to 419 ppm since the industrial revolution^1^, causing global warming (Lackner et al., 1995; Lal, 2008). Carbon dioxide removal technologies have been proposed to limit the mean global temperature increase to 1.5°C above preindustrial levels (Oelkers and Cole, 2008; Rogelj et al., 2016), and enhanced silicate weathering has been suggested as one of these solutions (Seifritz, 1990; Schuiling and Krijgsman, 2006; Hartmann et al., 2013). Silicate weathering is a natural process that can remove CO_2_ from the atmosphere on a geologically historical time scale (Cornwall et al., 2013). The removal of atmospheric CO_2_ could be stimulated with enhanced weathering of olivine (a type of silicate mineral) by its application to forests or oceans (Berner et al., 1983; Schuiling and Krijgsman, 2006). Since the industrial revolution, the ocean has absorbed one-third of the CO_2_ emitted by human activity (Sabine et al., 2004). The oceans have tremendous potential to remove CO_2_ and at less risk than does the land (Kheshgi, 1995; Kantola et al., 2017; Bach et al., 2019). Olivine added to seawater can enhance buffering capacity and absorb extra CO_2_ from the atmosphere. However, little is known about the impact of mineral dissolution products on microbes in the ecosystem, which plays a vital role in ocean carbon cycling (Meysman and Montserrat, 2017; Montserrat et al., 2017).

Microbial utilization and transformation of various forms of carbon are important regulators of global carbon fluxes (Jiao et al., 2010; Zhao et al., 2019). Bacteria generally can be characterized as particle-attached (PA) and free-living (FL) bacteria based on their different life strategies (Azam et al., 1983; Crump et al., 1999; Zhang et al., 2016). PA bacteria prefer to attach themselves to the suspended particulate matter and degrade parts of bioavailable particles into dissolved organic matter and inorganic nutrients supporting the surrounding biomass production (Ploug et al., 1999; Bachmann et al., 2018). The presence of suspended particles and its various quality can considerably influence the PA bacterial community (Smith et al., 2013). The addition of olivine powder to seawater not only releases ions into the surrounding seawater but also provides particles to attach to. However, the interaction between olivine particles and PA bacteria communities is not yet clear. Meanwhile, the accumulation of mineral dissolution products in the seawater might also influence the FL bacteria suspended in the water column (Meysman and Montserrat, 2017). In the context of global warming, the addition of olivine to seawater, without the effect of olivine on the bacteria, makes the current study very important.

In this study, industrial-grade olivine was added to natural coastal seawaters, and the bacterial community was investigated within 10 days of incubation. The goal of this study is to understand the following: (1) How do the PA and FL bacterial communities in the coastal seawater respond to the addition of olivine? (2) What type of mechanisms underpin the microbial response to the addition of olivine?

## Materials and Methods

### Experimental Procedure

**FIGURE 1:**
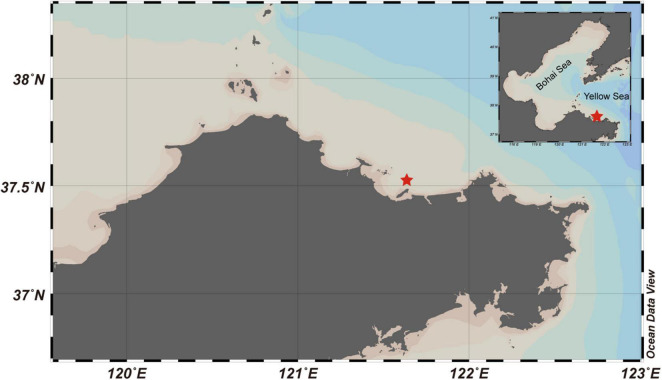
Map of Muping Marine Ranch (Yantai) with a star (★) showing the sampling area. Samples were collected from the surface and bottom of the sea on August 10, 2019.

The natural seawater used in this experiment was from the Muping Marine Ranch, Yantai, China (Figure 1), where the maximum depth is ∼22 m, with an averaged tidal current rate of ∼0.5 m s^–1^ (Lu et al., 2015; Li et al., 2021). In this ranch, the abundance and diversity of the bacterial community have been altered greatly by anthropogenic activities and aquaculture (Li et al., 2021). Before the experiment, the seawater was collected in the morning (8:00 to 10:00 a.m.) of August 10, 2019 from the surface (∼1-m depth) and bottom layers (∼1 m above the seabed) of the ranch with a Niskin bottle (Sea–Bird). The seawater collected from each depth was put into three 20-L dark buckets and transported to the laboratory within 10 h. After returning to the laboratory, the seawater from each layer was mixed, filtered with a 200-μm nytex net, and dispended into six 10-L transparent polycarbonate buckets (Nalgene), three of which were added with commercially available olivine powder to a final concentration of 1‰ (m/m). The remaining buckets were set as the control. The chemical composition of olivine powder includes MgO (35–50%), SiO_2_ (37–42%), ΣFe (≤10%), and CaO (<1%)^2^, and its particle size quantiles are D_10_ = 3.93 μm, D_50_ = 30.3 μm, and D_90_ = 133 μm, determined by a Mastersizer 3000 (Malvern, United Kingdom).

In total, 12 independent biological cultures were used in this experiment. To mimic the *in situ* condition, the surface layer of seawater with or without the addition of olivine powder was exposed to natural sunlight, and the bottom layer was kept in the dark by wrapping the buckets in aluminum foil. Temperature in the buckets was maintained at 25 ± 1.0°C (close to field condition) in a large thermostat-controlled bath. During the 10-day incubation, the buckets were manually mixed once a day to make the bacteria and particles suspend homogeneously. On day 0 (before olivine addition) and day 10 (the end of incubation), the seawater samples from each bucket were collected to determine the environmental variables and particle-attached and free-living bacteria compositions, as below.

### Dissolved Silicate, pH, and Total Alkalinity Measurements

Before seawater was collected at the sampling site, the site temperature and salinity were measured using a Water Quality Monitor (Seabird, United States). In the laboratory, on day 0 and day 10 of incubation, an aliquot of 250-ml seawater was taken from each bucket with or without olivine addition and filtrated with a syringe to avoid exposure to air. The filtration was collected to measure dissolved silicate (DSi) concentration, pH, and total alkalinity (TA).

For DSi measurement, duplicate 15-ml filtration samples were dispensed into 15-ml tubes and stored at −20°C for later analysis. The DSi was measured with an automatic nutrient analyzer (SEAL AA3, German) with the molybdate blue method (Hansen and Koroleff, 1999). The uncertainty in DSi measurements was ± 0.1 μmol kg^–1^.

For pH measurements, an aliquot of 40-ml filtration was immediately dispensed into a borosilicate glass without headspace. The pH was then measured with a Fisher pH-meter (Star A211) equipped with an Orion combined electrode (8157BNUMD). Before measuring, the pH meter was calibrated with NIST buffers (pH = 4.01, 7.00, 10.01 at 25°C). The uncertainty in pH measurements was ± 0.01.

For TA measurements, an aliquot of 120-ml filtration was dispensed into high-density polyethylene bottles, preserved with 0.02% saturated HgCl_2_ solution, and pre-stored in the dark at room temperature. The TA concentration was measured through Gran titration with an automatic titration system (T960, HANON). The certified reference materials (batch 178) from the Dickson lab were used for quality control. The uncertainty in TA measurements was ± 2 μmol kg^–1^.

### DNA Extraction and Sequencing

On day 0 and day 10, a 500-ml seawater sample was collected from each bucket with or without the olivine addition. The collected seawater was firstly filtrated through a 20-μm nylon sieve and then sequentially filtrated through 3- and 0.2-μm pore size polycarbonate membrane filters (47-mm diameter; Millipore) for microbe collection at a negative pressure of <0.01 MPa. The bacteria that settled on the membrane filter of 3-μm pore size were defined as the particle-attached fraction (PA, 3–20 μm), while those that settled on the filter of 0.2-μm pore size were defined as the free-living fraction (FL, 0.2–3 μm). The DNA of bacteria from both the 3- and 0.2-μm filters were extracted using a DNeasy PowerSoil Kit (Qiagen, Germany) according to the manufacturer’s protocol.

Amplicon sequencing of the microbial community in the DNA extraction was performed by Tianjin Novogene Bioinformatic Technology Co., Ltd. (Tianjin, China). The PCR products of one replicate of the olivine-added group in surface seawater are not enough for sequencing, so this part of the data is excluded from the data processing. The V3–V4 region of the bacterial 16S rDNA gene was amplified using the primer pair 341F (5′-CCTAYGGGRBGCASCAG-3′) and 806R (5′-GGACTACNNGGGTATCTAAT-3′) (Zhang et al., 2016), and sequencing was performed on an Illumina Hiseq 2500 pe250.

### Data Analysis

Sequences were denoised and clustered using amplicon sequencing variants (ASVs) according to the SILVA database (V138) using Qiime2 (Bolyen et al., 2019). All the sequences assigned to chloroplast and mitochondrion origins were removed from the dataset. Normalized rarefaction was performed, and all data were rarefied to 7,989 sequences per sample because sequencing depth influences diversity analysis. Statistical analyses were conducted in the R software (R Core Team, 2013). Alpha diversity was estimated with the Shannon–Weiner diversity index, and the significance of bacterial α-diversity was tested using Student’s *t*-tests. Principal coordinates analysis (PCoA) was carried out with a Bray–Curtis (BC) distance matrix using the “vegan” package, and information on chemical parameters was added to the PCoA plot using envfit to explore correlations between changes in bacterial community composition and environmental factors (Oksanen et al., 2020). Analysis of similarities (Anosim) using BC distance matrices was used to test the significance of the grouping based on the PCoA ordination. Similarity percentage analysis (SIMPER) was used to identify the major classes primarily responsible for the dissimilarity of bacterial community between control and olivine-added groups. Differences between control and olivine-added groups were analyzed by *t*-tests, and the confidence level was set at 0.05.

## Results

### Olivine Dissolution in Seawater

**FIGURE 2:**
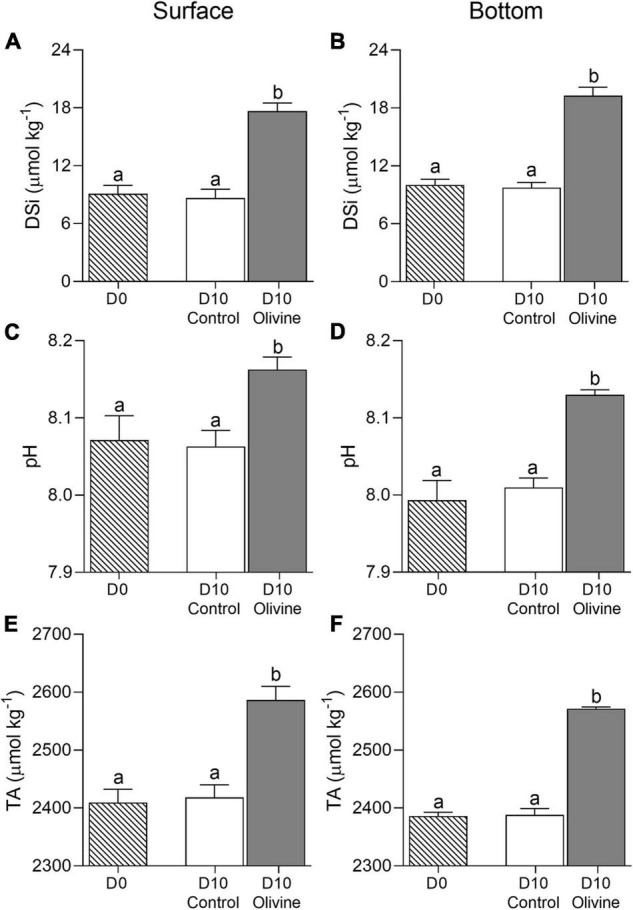
Changes of dissolved silicate concentration **(A,B)** DSi, μmol kg^–1^, pH **(C,D)**, and total alkalinity **(E,F)** μmol kg^–1^ in initial and 10-day incubated seawater with or without olivine addition, from the surface **(A,C,E)** and bottom layers **(B,D,F)** of the sampling site. The vertical bar indicates the standard deviation (*N* = 3), and different letters indicate significant differences (*p* < 0.05).

The temperature in the field was 25.2 ± 0.2°C and 24.6 ± 0.2°C in the surface and bottom layers of the sampling sites, respectively, while the salinity was 31.55 ± 0.16 and 31.78 ± 0.04, respectively. Accordingly, the initial DSi was 9.1 ± 0.9 and 10.0 ± 0.6 μmol kg^–1^, the pH was 8.07 ± 0.03 and 7.99 ± 0.03, and TA was 2410 ± 23 and 2386 ± 7 μmol kg^–1^, respectively (Figure 2). The temperature, TA, and pH in the surface layer were higher than in the bottom layer, while the salinity and DSi were lower in the surface layer.

The 10-day incubation caused no significant changes of seawater DSi, pH, and TA compared to the control for both the surface and bottom layers (Figure 2). In the olivine-added group, however, the DSi, pH, and TA were markedly increased in both layers (*p* < 0.01 in all cases). Adding the olivine increased the seawater DSi by 8.6 ± 0.1 μmol kg^–1^ from 9.1 ± 0.9 to 17.7 ± 0.8 μmol kg^–1^ in the surface water within 10 days of incubation, and by 9.3 ± 0.6 μmol kg^–1^ from 10.0 ± 0.6 to 19.2 ± 0.9 μmol kg^–1^ in the bottom water (Figures 2A,B). Meanwhile, the olivine addition increased the pH by 0.09 ± 0.02 in the surface (from 8.07 ± 0.03 to 8.16 ± 0.02) and by 0.14 ± 0.02 in the bottom (from 7.99 ± 0.03 to 8.13 ± 0.01) (Figures 2C,D), and increased the TA by 177 ± 4 μmol kg^–1^ (from 2410 ± 23 to 2587 ± 24 μmol kg^–1^) and 185 ± 4 μmol kg^–1^ (from 2386 ± 7 to 2572 ± 3 μmol kg^–1^), respectively (Figures 2E,F).

### Response of the Bacterial Community to Olivine Addition

**FIGURE 3:**
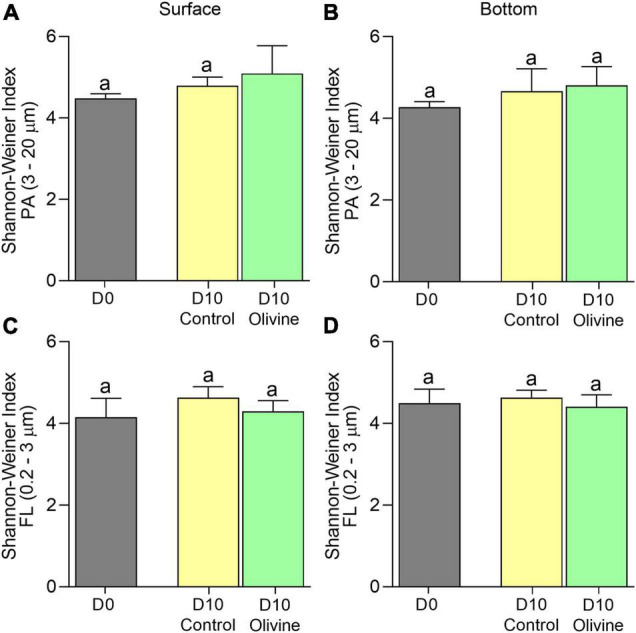
Changes of Shannon–Weiner index of the particle-attached **(A,B)** PA, 3–20 μm and free-living **(C,D)** FL, 0.2–3 μm bacterial communities in initial and 10-day incubated seawater with or without olivine addition, from the surface **(A,C)** and bottom layers **(B,D)** of the sampling site. The vertical bar indicates the standard deviation (*N* = 3), and different letters indicate significant differences (*p* < 0.05).

The PA bacterial diversity and richness of the control groups showed a small range of variation over time (4.23–4.39 in surface seawater incubation and 3.82–4.28 in bottom seawater incubation). That of the olivine-added groups increased slightly (4.23–4.86 in surface seawater incubation and 3.82–4.59 in bottom seawater incubation) (Figures 3A,B). Similar to the PA bacteria, the FL bacterial diversity and richness of both the control and olivine-added groups changed little over time (Figures 3C,D). Moreover, there were no significant differences in bacterial diversity and richness between the two groups after 10 days of incubation for both FL and PA fractions according to the *t*-test (*p* > 0.05).

Variation in the bacterial community composition was evaluated by using PCoA of the Bray–Curtis distance. Analysis indicated the distinctiveness of the PA bacterial community after olivine addition (Supplementary Figures 1A,B). The first two constrained axes together explained 47.34 and 51.49% of the variation in the PA bacterial community in the surface and bottom water incubations, respectively. The bacterial communities of the control and olivine-added groups were separated along the chemical properties after 10 days of incubation (*r*_*Anosim*_ = 0.58 in surface seawater; *r*_*Anosim*_ = 0.37 in bottom seawater). The two PCoA axes, PCo1 and PCo2, captured 36.1 and 33.26%, and 17.74 and 21.79% of the total variation in the FL bacterial community in the surface and bottom incubations, respectively. The changes of bacterial community composition over time were primarily separated along PCo1 (Supplementary Figures 1C,D). The FL bacterial community showed a visible differentiation between day 0 and day 10, but the FL bacterial community of the olivine-added group on day 10 was not separated from the control group, which clustered together (*r*_*Anosim*_ = 0.07 in surface seawater; *r*_*Anosim*_ = −0.07 in bottom seawater).

**FIGURE 4:**
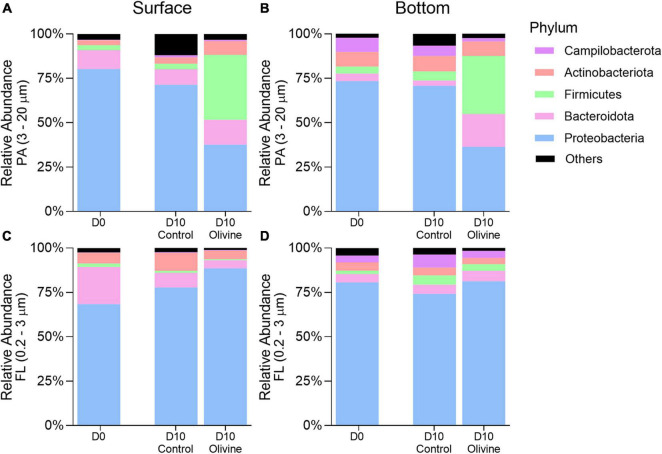
Changes of averaged relative abundance (*N* = 3) of particle-attached **(A,B)** PA, 3–20 μm and free-living **(C,D)** FL, 0.2–3 μm bacterial communities at the phylum level in initial and 10-day incubated seawater with or without olivine addition, from the surface **(A,C)** and bottom layers **(B,D)** of the sampling site. The standard deviations of bacterial relative abundances are shown in Supplementary Figure 2.

The dataset consisted of 29 phyla, of which *Proteobacteria*, *Bacteroidota*, *Firmicutes*, *Actinobacteriota*, and *Campilobacterota* were the most abundant. Each of these five phyla accounted for ≥1% of the total sequences (all FL and PA libraries together) (Figure 4). *Proteobacteria* was the most abundant phylum in both PA and FL bacterial communities in the control groups on day 10. The sequences affiliated to *Bacteroidota* and *Firmicutes* in the PA fraction responded differently to the addition of olivine compared with the control in both the surface and bottom seawater incubations after 10 days. In the surface seawater, the relative abundance of *Firmicutes* increased considerably after adding olivine, and the distribution of the *Bacteroidota* sequences did not vary much between the control and olivine-added groups. In the bottom seawater, *Bacteroidota* and *Firmicutes* became the dominant bacteria, comprising more than 50% of the bacterial community after olivine addition. Five major phyla of the FL fraction showed a non-significant difference between the control and olivine-added groups, and *Proteobacteria* was the dominant phylum in both groups. The distributions of *Bacteroidota*, *Firmicutes*, and *Actinobacteriota* in the control were similar to those in the olivine-added group.

**FIGURE 5:**
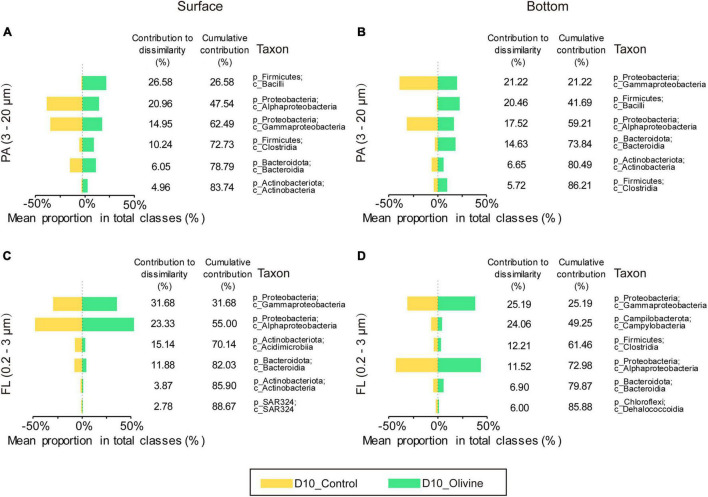
Contributions of the top six particle-attached **(A,B)** PA, 3–20 μm and free-living **(C,D)** FL, 0.2–3 μm bacteria classes to community dissimilarities after 10-day incubations in control and olivine-added seawater from surface **(A,C)** and bottom layers **(B,D)** of the sampling site.

**FIGURE 6:**
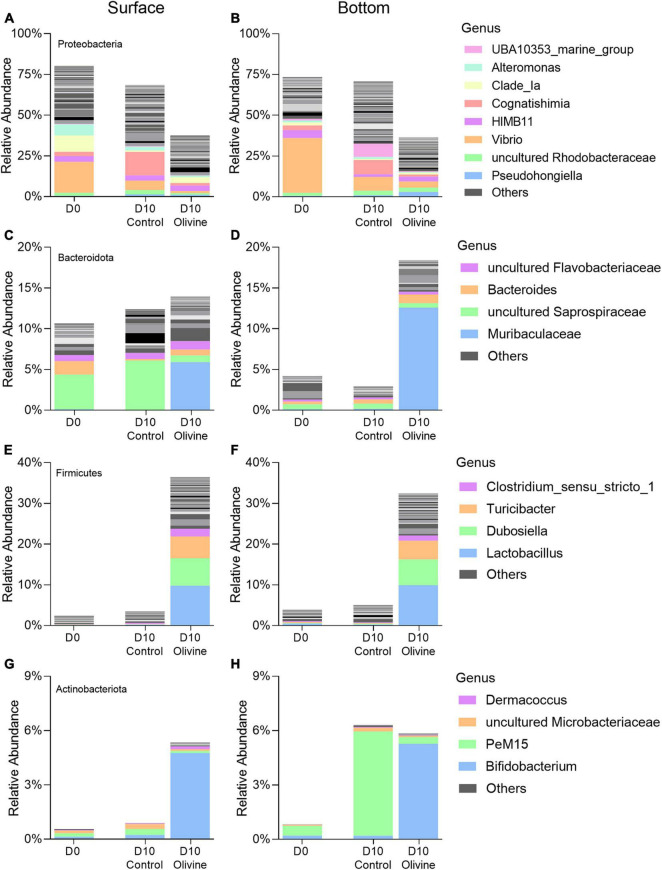
Changes of averaged relative abundance (*N* = 3) of particle-attached (PA, 3–20 μm) bacterial communities that respond to olivine addition at the genera level, in initial and 10-day incubated seawater with or without olivine addition: **(A,B)** *Proteobacteria* (*Alphaproteobacteria* and *Gammaproteobacteria*); **(C,D)** *Bacteroidota* (*Bacteroidia*); **(E,F)** *Firmicutes* (*Bacilli* and *Clostridia*); and **(G,H)** *Actinobacteriota* (*Actinobacteria*) in the surface **(A,C,E,G)** and bottom layers **(B,D,F,H)** of the sampling site. The standard deviations of bacterial relative abundances are shown in Supplementary Figure 2.

Similarity percentage analysis analysis identified six classes primarily responsible for the observed bacterial community dissimilarities of the PA fraction between the control and olivine-added groups (Figures 5A,B), and these classes contributed more than 85% to the overall dissimilarities. In surface seawater, of all the six classes, two classes of *Firmicutes* contributed the most to the overall dissimilarities (36.82%), followed by *Proteobacteria* (two classes, 35.91%), *Bacteroidota* (one class, 6.05%), and *Actinobacteriota* (one class, 4.96%). The relative abundances of classes affiliated with *Firmicutes* were much higher in the olivine-added group (12.0–24.4%) than the control group (0.6–2.8%) (Figure 5A). The observed differences in bottom seawater were similar to those in surface seawater, and the olivine-added group in bottom seawater incubation was characterized by high relative abundances of *Bacilli*, *Clostridia* (affiliated with *Firmicutes*), and *Bacteroidia* (affiliated with *Bacteroidota*). For the FL bacterial community, in surface seawater, *Gammaproteobacteria* and *Alphaproteobacteria* were the two most different classes, making up more than half of the observed differences, followed by *Acidimicrobiia*, *Bacteroidia*, *Actinobacteria*, and *SAR324* (Figure 5C). In bottom seawater, the differences in the FL bacterial community between the control and olivine-added groups were mainly affected by *Gammaproteobacteria*, *Campylobacteria*, *Clostridia*, *Alphaproteobacteria*, *Bacteroidia*, and *Dehalococcoidia* (Figure 5D). Further analysis of the genera from the classes of PA bacteria that responded to olivine addition showed that the members of *Lactobacillus*, *Dubosiella*, and *Turicibacter* from class *Bacilli* and *Clostridium sensu stricto 1* from class *Clostridia* were more abundant after olivine addition (Figures 6E,F). Meanwhile, *Bifidobacterium* (class *Actinobacteria*) and *Muribaculaceae* (class *Bacteroidia*) were enriched in the olivine-added groups (Figures 6C,D,G,H).

## Discussion

Olivine weathering in seawater can effectively enhance the alkalinity of the seawater (Montserrat et al., 2017). Consistent with previous studies (Hartmann and Kempe, 2008; Hangx and Spiers, 2009; Renforth and Henderson, 2017), the addition of olivine in this experiment resulted in a significant increase in pH, TA, and DSi concentrations relative to the control groups in both surface and bottom seawater incubations (Figure 2). Previous studies indicated that the accumulation of dissolution products and particles might influence the bacterial community (White and Brantley, 2003; Meysman and Montserrat, 2017). However, until now the mechanisms behind this have not been clear.

We further explored the variations in the bacterial community in seawater after adding olivine through the laboratory culture experiment of natural surface and bottom seawater. PA and FL bacterial communities responded differently to olivine addition after 10 days of incubation (Supplementary Figure 1). The PCoA and analysis of similarities (Anosim) indicated that the addition of olivine had no evident influence on the FL bacterial community during the experimental period, which might be due to the fact that coastal FL bacteria have adapted to the *in situ* near-shore environmental fluctuations (Cornwall et al., 2013; Jing et al., 2013; Liu et al., 2019), and they were not attached to the olivine particles nor exposed to the microenvironment formed by olivine dissolution. However, the analysis of similarities suggested that PA bacterial communities of the control and olivine-added groups were different after 10 days of incubation. The interaction between olivine particles and biofilm-forming microbes was considered the main reason for changes in PA bacterial community composition (Shirokova et al., 2012; Caraballo Guzman et al., 2020). Phyla *Bacteroidota* and *Firmicutes* have an inclination for growth attached to particles (Fernandez-Gomez et al., 2013; Zhang et al., 2016; Bachmann et al., 2018). After grinding, olivine has a large specific surface area and that of ground olivine provides more opportunities for bacteria to colonize when olivine particles float in the seawater column (Smith et al., 2013; Rigopoulos et al., 2015). Similar to our findings, a high proportion of *Bacteroidota* was also found on the glass beads (Ogonowski et al., 2018), indicating that *Bacteroidota* can not only attach to polymers but also colonize inorganic particles. Members of *Bacilli*, *Clostridia*, and *Bacteroidia* were enriched in the PA fraction after olivine addition. Previous studies indicated that biofilm formation by *Bacilli* conferred antibiotic resistance, and *Clostridia* could be found in moving bed biofilm reactor systems (Houry et al., 2012; Pantaleon et al., 2014). *Bacteroidia* was identified as the core microbiome of the polyethylene-associated biofilm in coastal environments (Tu et al., 2020). The high concentrations of mineral dissolution products around the particles might stimulate microorganisms to facilitate biofilm development to resist environmental stress (Negrete-Bolagay et al., 2021). The complex structure of biofilms might lead to diffusion limitation, and the oxygen distribution was strongly influenced by the biofilm depth. As a result, the anaerobic area formed at some depth below the biofilm surface, which could facilitate the cohabitation of anaerobic and aerobic bacteria within the microbial biofilm (Tay et al., 2002). Strictly or facultative anaerobic bacterial genera were enriched in the PA fraction during the 10-day incubation, indicating the possible formation of an anoxic microenvironment (Figure 6). *Lactobacillus*, one of the major PA bacterial taxa in olivine-added groups, was a genus of facultative anaerobic bacteria that could grow as biofilms on abiotic surfaces and was enriched in the PA fraction of all samples. *Bifidobacterium* was a strictly anaerobic genus, which was enriched in the olivine-added groups, and bifidobacterial biofilm formation might be a multifactorial adaptive phenomenon in response to olivine exposure (de Vries and Stouthamer, 1969; Liu et al., 2021). The anaerobic genera *Muribaculaceae*, *Dubosiella*, and *Turicibacter* were also enriched after olivine addition in the PA fraction, indicating that micro-anaerobic conditions might form (Licht et al., 2007; Liu et al., 2020; Park et al., 2021).

The residence time of olivine in the seawater column mainly depends on the size of the particles (Hangx and Spiers, 2009). Smaller-size olivine particles remain in the surface layer for longer. The quality of suspended matter also influences microbial colonization. It has been reported that the bacterial abundance on organic and aged particles is higher than that on inorganic and fresh particles (Kernegger et al., 2009). Inversely, the secondary production of individual bacteria on inorganic and fresh particles is higher than that on organic and aged ones (Kernegger et al., 2009). At the same time, the attachment and remineralization of organic particulate matter by PA bacteria can retain nutrients and DOM in the surface layer, which is important in summer when the thermocline forms and nitrogen and phosphorus nutrients are limited (Simon et al., 2002; Hangx and Spiers, 2009). Meanwhile, olivine dissolution enriches the DSi pool in the surface ocean (Bach et al., 2019). The accumulated nutrients can stimulate planktonic algal blooms to take up more CO_2_ if olivine addition conducts in a reasonable time.

## Conclusion

The dissolution of olivine can effectively raise the alkalinity and pH of seawater, and thus uptake extra CO_2_ from the atmosphere, and mitigate ocean acidification. Our study showed that the influence of olivine addition on the bacterial community was mainly on particle-attached bacteria rather than the free-living bacterial community during the experimental period. The difference between the particle-attached bacterial community in the control and olivine-added groups might be due to environmental stress resulting from the olivine dissolution, which could stimulate particle-attached bacteria to facilitate biofilm development. Further investigation on ecological effects is still needed before large-scale enhanced weathering through the dissolution of olivine, even though it is feasible and effective.

## Data Availability Statement

The datasets presented in this study can be found in online repositories. The names of the repository/repositories and accession number(s) can be found below: https://www.ncbi.nlm.nih.gov/, PRJNA755193.

## Author Contributions

YH designed the research. HR, LM, and ZZ conducted the experiments. HR analyzed the data and drafted the manuscript. NJ, YH, JL, GL, QZ, YP, and HR discussed the interpretation of the results. All authors have agreed to authorship and have approved the manuscript submission.

## Conflict of Interest

The authors declare that the research was conducted in the absence of any commercial or financial relationships that could be construed as a potential conflict of interest.

## Publisher’s Note

All claims expressed in this article are solely those of the authors and do not necessarily represent those of their affiliated organizations, or those of the publisher, the editors and the reviewers. Any product that may be evaluated in this article, or claim that may be made by its manufacturer, is not guaranteed or endorsed by the publisher.

## Footnotes

1 https://gml.noaa.gov/ccgg/trends/ (accessed May 2021).

2
http://www.fqgls.com/about_t_2.html
