# Supplementary material for: Response of a Coastal Microbial Community to Olivine Addition in the Muping Marine Ranch, Yantai
Source: Front Microbiol. 2022 Feb 10;12:805361. doi: 10.3389/fmicb.2021.805361 (PMC8867022; doi:10.3389/fmicb.2021.805361)
Supplement: Supplementary file 1 [file Data_Sheet_1.docx]

Supplementary Material

## Supplementary Figures

**
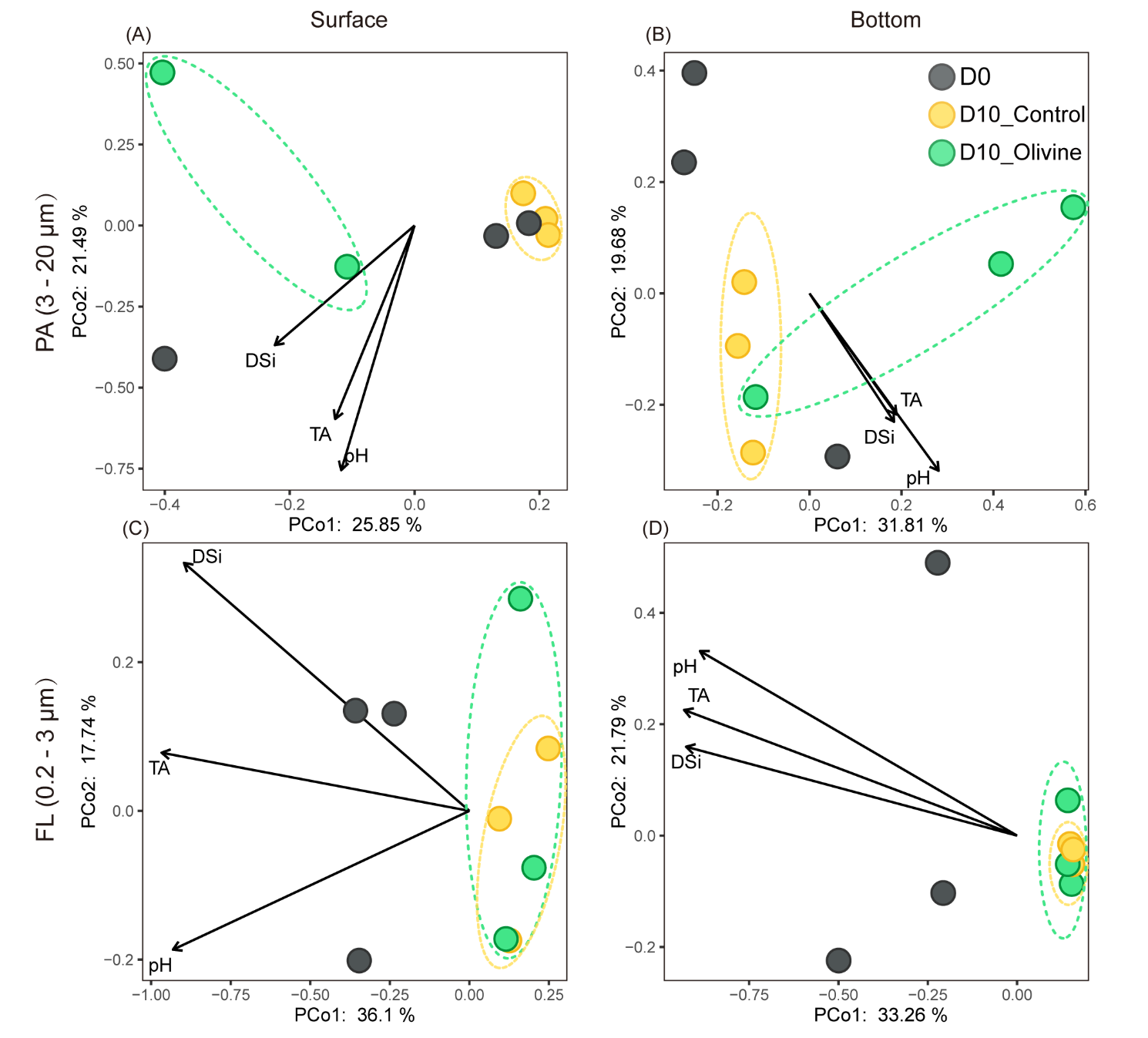
**

**Figure S1**. Principal coordinates analysis (PCoA) for particle-attached (**A**, **B**; PA, 3-20 µm) and free-living (**C**, **D**; FL, 0.2-3 µm) bacterial community compositions in seawater from surface (**A, C**) and bottom (**B, D**) layers of sampling site. Information on chemical parameters was added to the PCoA plot using envfit.


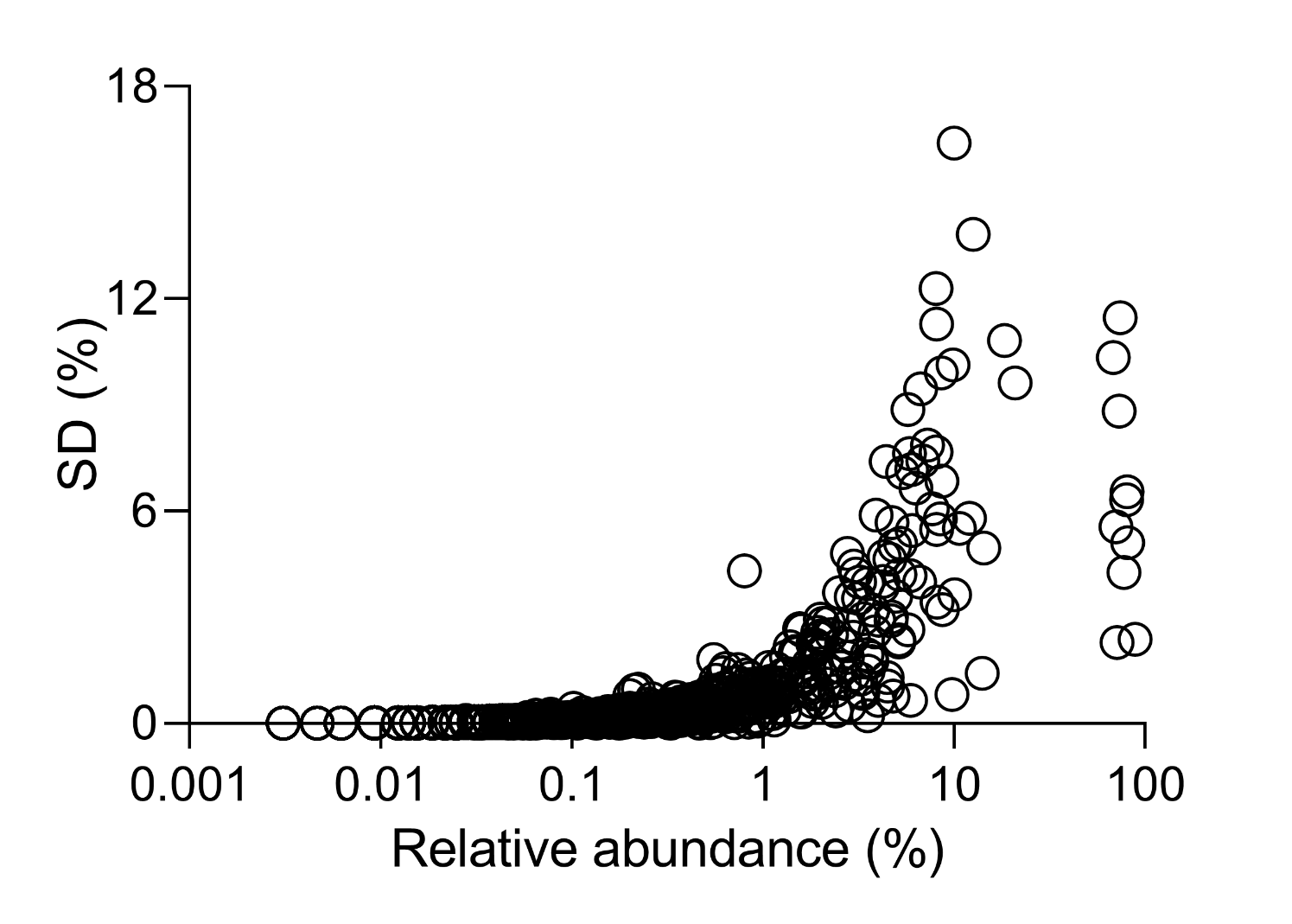


**Figure S2**. Standard deviations (SD, %) against relative abundance (%) of different bacterial communities to indicate the variation of three independently biological measurements.
